# Supplementary material for: Emergency capacity analysis in Ethiopia: Results of a baseline emergency facility assessment
Source: PLoS One. 2022 Jan 21;17(1):e0258310. doi: 10.1371/journal.pone.0258310 (PMC8782317; doi:10.1371/journal.pone.0258310)
Supplement: S1 Appendix — (DOCX) [file pone.0258310.s001.docx]

**Appendix**

**Figure I: Triage Equipment Availability**


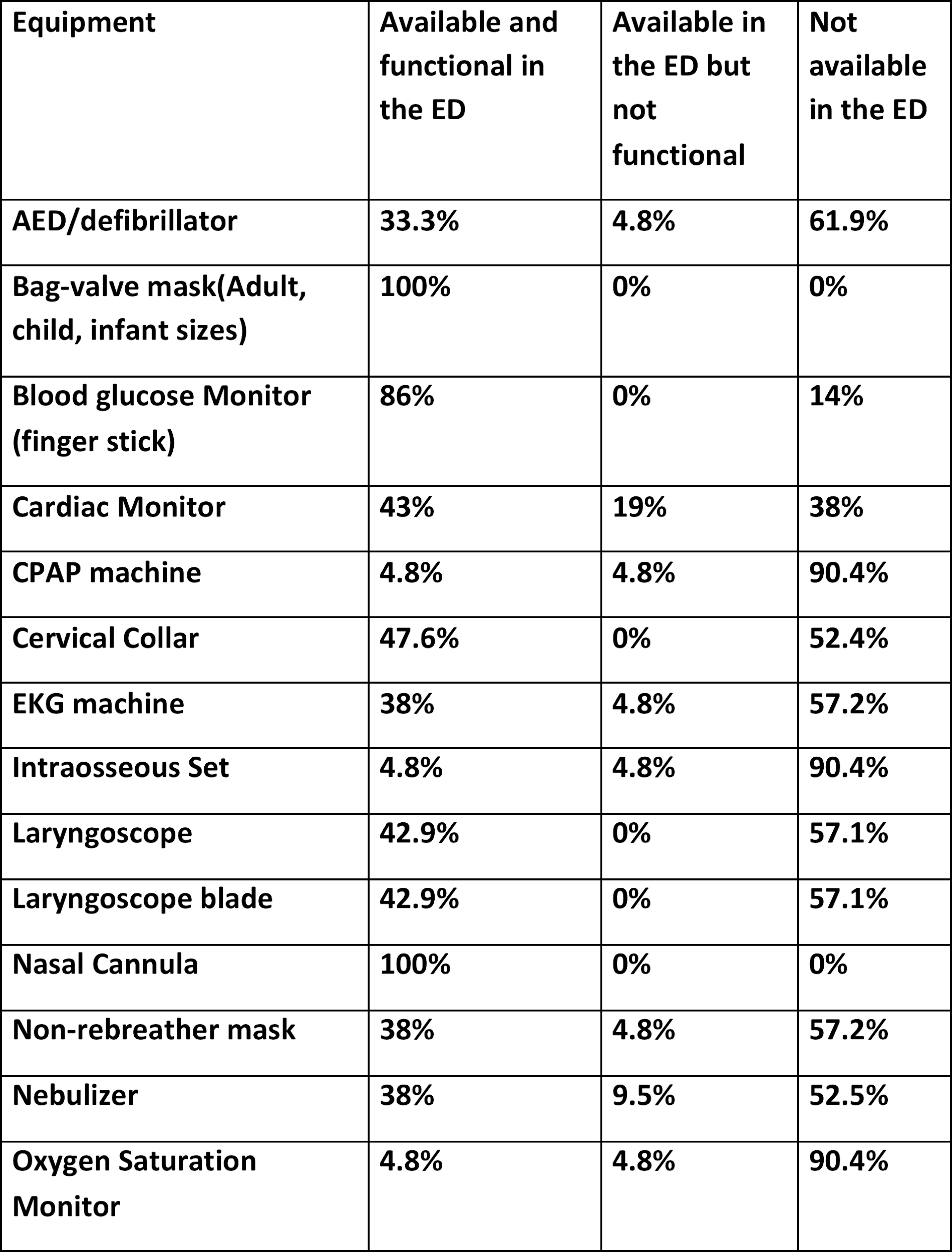


**Figure II: Essential Emergency Equipment**

| **Medication** | **% Hospitals with Access** |
| --- | --- |
|  |  |
| **Asthma/COPD** |  |
| Oral steroids | 54.55% |
| IV steroids | 36.36% |
| Salbutamol | 90.91% |
|  |  |
| **RSI/Cardiac Arrest** |  |
| IV paralytics | 27.27% |
| Adrenaline 1:1000 | 86.36% |
| Adrenaline 1:10,000 | 45.45% |
|  |  |
| **Volume Resuscitation** |  |
| Oral rehydration therapy | 95.45% |
| IV fluids | 59.09% |
| Transfusion products (whole blood, FFP, pRBC) | 31.82% |
|  |  |
| **Glucose Control (IV/ oral)** |  |
| 10% dextrose | 9.09% |
| Dextrose 40 or 50% injection | 95.45% |
| Insulin | 90.91% |
|  |  |
| **ACS** |  |
| Aspirin | 90.91% |
| Nitroglycerine | 22.73% |
| Thrombolytic (alteplase, streptokinase) | 0.00% |
|  |  |
| **Toxin Ingestion** |  |
| Activated charcoal | 9.09% |
| Narcan (naloxone) | 18.18% |
| Sodium bicarbonate | 31.82% |
| Atropine | 95.45% |
| Antivenom (snake) | 40.91% |
| Vitamin K (PO) | 0.00% |
| Vitamin K (IV) | 86.36% |
|  |  |
| **Diuretics ( PO /IV)** |  |
| furosemide | 90.91% |
|  |  |
| **Rate Control Medications** |  |
| Beta-blocker (IV) | 54.55% |
| Calcium channel blockers (IV) | 9.09% |
|  |  |
| **Sedation ( PO/IV)** |  |
| Haloperidol IV or IM | 72.73% |
| Benzodiazepin | 0.00% |
| Diazepam | 90.91% |
|  |  |
| **Analgesics** |  |
| narcotic (IV) | 63.64% |
| NSAID (IV) | 72.73% |
| Local anesthesia | 0.00% |
| lidocaine (without adrenaline) | 81.82% |
| lidocaine with adrenaline | 45.45% |
| Paracetamol | 95.45% |
|  |  |
| **OBG** |  |
| Antenatal steroids | 0.00% |
| Magnesium sulphate | 0.00% |
| Magnesium | 86.36% |
| Oxytocin | 0.00% |
| Pyridoxine | 81.82% |
| Anti-D IG | 54.55% |
|  |  |
| **Infectious Disease (PO/IV)** |  |
| Metronidazole | 86.36% |
| Ceftriaxone | 90.91% |
| Amoxicillin/Clavulanic acid | 36.36% |
| Gentamicin | 50.00% |
| Ciprofloxacin | 40.91% |
| Clindamycin | 22.73% |
| Vancomycin | 31.82% |
| beta-lactam antibiotic (any oral) | 54.55% |
| Topical antifungals | 0.00% |
| Oral antifungals | 0.00% |
| Antimalarials (PO/IV) | 0.00% |
| Anthelmintics | 0.00% |
| Antifungals (PO/IV) | 0.00% |
| Topical antibacterials | 0.00% |
| Topical steroids | 0.00% |
| Tetanus Vaccine | 0.00% |
| Rabies vaccine | 31.82% |
| Antirabies IG | 0.00% |
|  |  |
| **Antiemetics ( PO/IV)** |  |
| Metoclopramide | 95.45% |
|  |  |
| **Other** |  |
| Mannitol | 68.18% |
| PTU | 36.36% |
| Potassium chloride | 95.45% |

| **Procedural Equipment** | **% Hospitals with Access** |
| --- | --- |
| Cervical Spine Immobilization | 45.45% |
| Oral airway placement | 68.18% |
| Nasal airway placement | 27.27% |
| Endotracheal intubation | 45.45% |
| Surgical airway placement | 0.00% |
| BMV ventilation | 95.45% |
| O2 administration | 95.45% |
| Needle decompression | 0.00% |
| Chest drain placement | 40.91% |
| IV fluid infusion | 86.36% |
| Defibrillation/ cardioversion | 36.36% |
| Mechanical ventilation | 9.09% |
| NPPV | 0.00% |
| Central line placement | 4.55% |
| Pericardiocentesis | 9.09% |
| Pacing | 0.00% |
| Rewarming (active/passive) | 0.00% |
| Lumbar Puncture | 40.91% |
| Suturing laceration | 86.36% |
| Fasciotomy/ Escharotomy | 0.00% |
| Closed reduction & casting | 54.55% |
| Joint reduction | 0.00% |
| Placement of external fixator | 54.55% |
| Use of traction | 0.00% |
| Splinting | 4.55% |
| Abscess drainage | 0.00% |
| Wound care | 0.00% |
| Urinary catheterization | 77.27% |
| Suprapubic cystostomy | 0.00% |
| Procedural sedation (etomidate) | 0.00% |
| Regional block | 0.00% |
| Restraint | 31.82% |
| Nasogastric tube | 77.27% |

Figure III: Summary of Resuscitation Standards Established by Ethiopian Standard Agency

| Requirements | Hospital Level | | |
| --- | --- | --- | --- |
|  | Primary^8^ | Secondary^9^ | Tertiary^10^. |
| Personnel Training | | | |
| CPR | ✓ | ✓ | ✓ |
| Procedures | | | |
| BLS: CPR, airway management and/or oxygen supply, hemorrhage control, fluid resuscitation | ✓ | ✓ | ✓ |
| Newborn resuscitation | ✓ | ✓ | ✓ |
| Cardiac Resuscitation: cardiac arrest, malignant ventricular arrhythmia, sudden life threatening rhythm disturbances and congenital heart disease, cardiogenic shock, |  |  | ✓ |
| Infrastructure | | | |
| Designated resuscitation area for:  -neonates in in labor and delivery ward, | ✓ | ✓ | ✓ |
| -children in pediatric ward |  | ✓ | ✓ |
| -cardiac services |  |  | ✓ |
| Resuscitation kits available in:  -emergency room, inpatient, outpatient,  -labor and delivery, anesthesia store,  -nursing care | ✓ | ✓ | ✓ |
| -surgical and orthopedic care,  -pediatrics ward, radiology |  | ✓ | ✓ |
| Resuscitation trolley available in:  -Anesthesia and ER | ✓ | ✓ | ✓ |
| -ICU, ENT |  | ✓ | ✓ |
| Crash cart available in OBG nurses station |  | ✓ | ✓ |
