# Supplementary material for: Emergency capacity analysis in Ethiopia: Results of a baseline emergency facility assessment
Source: PLoS One. 2022 Jan 21;17(1):e0258310. doi: 10.1371/journal.pone.0258310 (PMC8782317; doi:10.1371/journal.pone.0258310)
Supplement: Attachment — Submitted filename: Response to Reviewers - 3.docx [file pone.0258310.s004.docx]

Dear Reviewers,

Please see the following issues addressed:

1. Please amend your Response to Reviewers letter to include a point by point response to each of the points made by the Editor and / or Reviewers. Please follow this link for more information: <https://urldefense.proofpoint.com/v2/url?u=http-3A__blogs.PLOS.org_everyone_2011_05_10_how-2Dto-2Dsubmit-2Dyour-2Drevised-2Dmanuscript_&d=DwIGaQ&c=lb62iw4YL4RFalcE2hQUQealT9-RXrryqt9KZX2qu2s&r=umxSEMkcGadXDYDQmHtEVMzzj6hKkX9DmS7wSyJSuqo&m=FMEgiVO2HMjZmZldL5La-WmvZ9v7w5ewBqZDZbnKAlc&s=EF5q610FancagswuIC4I4pKko1drjC14ITtNc99UcrM&e=>

The following format has now been used.

2. Please amend the title either on the online submission form or in your manuscript so that they are identical.

The title has been revised.

3. We note you have included a table to which you do not refer in the text of your manuscript. Please ensure that you refer to Table IIIa and IIIb in your text; if accepted, production will need this reference to link the reader to the Table.

There is a figure III that has been referred to in line 277 – but no other tables/charts can be found that have not been referred to? Please let us know if anything else is being referred to.

4. If possible, please upload a file showing your changes either highlighted or using track changes.  This should be uploaded as a Revised Manuscript w/tracked changes, file type. Please follow this link for more information: <https://urldefense.proofpoint.com/v2/url?u=http-3A__blogs.PLOS.org_everyone_2011_05_10_how-2Dto-2Dsubmit-2Dyour-2Drevised-2Dmanuscript_&d=DwIGaQ&c=lb62iw4YL4RFalcE2hQUQealT9-RXrryqt9KZX2qu2s&r=umxSEMkcGadXDYDQmHtEVMzzj6hKkX9DmS7wSyJSuqo&m=FMEgiVO2HMjZmZldL5La-WmvZ9v7w5ewBqZDZbnKAlc&s=EF5q610FancagswuIC4I4pKko1drjC14ITtNc99UcrM&e=>

This file has been uploaded.

We appreciate all of the reviewer’s comments and look forward to discussing our paper further as needed. Please let us know if any further changes need to be made.

The Authors
